# Mixture Deconvolution with Massively Parallel Sequencing Data: Microhaplotypes Versus Short Tandem Repeats

**DOI:** 10.3390/genes16091105

**Published:** 2025-09-18

**Authors:** Monica Giuffrida, Pedro Rodrigues, Zehra Köksal, Carina G. Jønck, Vania Pereira, Claus Børsting

**Affiliations:** Section of Forensic Genetics, Department of Forensic Medicine, Faculty of Health Sciences, University of Copenhagen, 11 Frederik V’s Vej, DK-2100 Copenhagen, Denmark; monicagiuffrida@sund.ku.dk (M.G.); pedro.rodrigues@sund.ku.dk (P.R.); zehra.koksal@liu.se (Z.K.); carina.joenck@sund.ku.dk (C.G.J.); vania.pereira@sund.ku.dk (V.P.)

**Keywords:** forensic genetics, MPSproto, probabilistic genotyping software, next-generation sequencing, microhaplotype, short tandem repeat

## Abstract

**Background/Objectives:** Interpretation of mixture profiles generated from crime scene samples is an important element in forensic genetics. Here, a workflow for mixture deconvolution of sequenced microhaplotypes (MHs) and STRs using the probabilistic genotyping software MPSproto v0.9.7 was developed, and the performance of the two types of loci was compared. **Methods**: Sequencing data from a custom panel of 74 MHs (the MH-74 plex) and a commercial kit with 26 autosomal STRs (the ForenSeq™ DNA Signature Prep Kit) were used. Single-source profiles were computationally combined to create 360 two-person and 336 three-person mixtures using the Python script MixtureSimulator v1.0. Additionally, 72 real mixtures typed with the MH-74 plex and 18 real mixtures typed with the ForenSeq Kit from a previous study were deconvoluted using MPSproto. **Results**: The deconvoluted MH profiles were more complete and had fewer wrong genotype calls than the deconvoluted STR profiles. The contributor proportion estimates were more accurate for MH profiles than for STR profiles. Wrong genotype calls were mostly caused by locus and heterozygous imbalances, noise reads, or an inaccurate contributor proportion estimation. The latter was especially problematic in STR sequencing data, when two contributors contributed equally to the mixture. A total of 34,800 deconvolutions of the simulated mixtures were performed with two defined hypotheses: H_p_, “The sample consists of DNA from one/two unknown contributor(s) and the suspect” and H_d_, “The sample consists of DNA from two/three unknown individuals”. All true contributors were identified (LR > 10^15^ for MHs and LR > 10^9^ for STRs) and all non-contributors excluded (LR < 10^−6^ for MHs and LR < 0.2 for STRs). **Conclusions**: In simulated and real mixtures, the MHs performed better than STRs.

## 1. Introduction

For more than three decades, short tandem repeats (STRs) have been the preferred loci for human identification and kinship testing in forensic genetics [1,2]. Standard panels of STR loci are available [3] and used by forensic laboratories around the world, which allows for comparison of STR profiles between countries. More recently, microhaplotypes (MHs) were suggested as alternatives to STRs [4]. MHs are defined as short regions (200–300 bp) with two or more SNPs that may be amplified by PCR and efficiently sequenced using massively parallel sequencing (MPS). STRs and MHs are both multiallelic and highly polymorphic loci, which makes them ideally suited for individual identification [5,6]. However, sequenced MHs have two important advantages compared to sequenced STRs: (1) amplification of MHs does not generate stutter artefacts, and (2) the amplicon lengths of different MH alleles are the same. The former simplifies data analyses, and the latter prevents MPS read count variation due to differently sized alleles, which is observed for most STRs [7]. Thus, the interpretation of sequencing results should be simpler with MHs compared to STRs, especially for samples with DNA from more than one contributor [5].

There have been several reports on mixture interpretation using MH loci [8,9,10,11,12,13,14,15,16,17]. In most of these studies, the number of observed MH alleles was counted and compared to the known MH profiles in the mixtures, and in some studies, these numbers were also compared to the number of observed STR alleles detected with either capillary electrophoresis [8,9,12,16] or MPS [9] in the same mixtures. Overall, a higher percentage of alleles from the minor contributor(s) was detected in MH assays than in STR assays, and at lower contributor proportions. Often, STR alleles from the minor contributor(s) were indistinguishable from stutter artefacts of alleles from the major contributor and thus not identified. In contrast, complete or almost complete MH profiles from minor contributors were observed in 1:40 and 1:19 two-person mixtures [8,9,12]. Obviously, these observations depended on DNA input [8,9,17], criteria for identification of alleles [13,16], efficiency of the MH assay, and effective number of alleles (Ae) of the individual MH loci [14]. In three recent studies of mixtures, a probabilistic genotyping software was used to estimate the number of contributors in the mixture [16] and deconvolute the MH profile [15,16,17]. With the R package KinMix [18], it was possible to calculate the evidential weight and assess whether the same individual contributed to two different mixtures and whether close relatives were present in different mixtures [15] using a panel of 140 MHs [11]. EuroForMix [19] was used to evaluate whether an individual contributed to a mixture with two, three, or four people [16] and, based on the evidential weights, a panel of 118 short MHs [20] outperformed the GlobalFiler PCR Amplification Kit with 22 STRs. A truncated Gaussian model, accounting for allele drop-out, noise, and locus-specific detection efficiency, was developed by Wang et al. [17] for the deconvolution of MH mixture profiles. The model was validated on two- and three-person mixtures, yielding higher likelihood ratios (LRs) than EuroForMix [19] with no false negative results.

The aim of the present work was to (1) establish a uniform workflow for deconvolution of mixtures that were either sequenced for MHs or STRs, and (2) evaluate which set of loci was best suited for mixture interpretation. For this purpose, sequencing data generated with the Ion AmpliSeq™ MH-74 Plex Research Panel [21] and the ForenSeq™ DNA Signature Prep Kit [7,22] were analysed with MHinNGS [23] and STRinNGS [24], respectively, and deconvoluted using the probabilistic genotyping software MPSproto [25]. MPSproto was originally developed for the deconvolution of STR mixtures analysed with MPS. In this study, the software was successfully adapted for the deconvolution of MH mixtures. Simulated MH and STR mixtures were generated with the script MixtureSimulator v1.0 (https://github.com/dk-cph-ri-rga/MixtureSimulator) from single-source FASTQ files. A total of 180 two-person and 168 three-person mixtures were simulated for the two assays and further investigated with the aid of MPSproto. Additionally, a total of 72 and 18 mixtures generated in the laboratory were amplified with the MH-74 plex and the ForenSeq Kit, respectively, and deconvoluted with MPSproto.

## 2. Materials and Methods

### 2.1. Samples

Anonymous blood samples from six Danish individuals, two females and four males, were selected from the “Section of Forensic Genetics anonymous collection of samples” (ref. no. 004-0065/21-7000) and used to generate 36 two-person mixtures (Supplementary Table S1) in duplicate.

DNA extraction was performed using the QIAamp^®^ DNA Blood Mini Kit (250) (Qiagen, Hilden, Germany). Quantification of the samples was performed using the Qubit™ dsDNA HS Assay Kit (Thermo Fisher ScientificWaltham, MA, USA) and the Qubit™ 3.0 Fluorometer (Thermo Fisher Scientific).

In addition, sequencing data from previous work (Hussing et al. [7,22] and Tomas et al. [21]) were used for the current study. A total of 20 profiles were utilized to generate the 360 two-person and the 336 three-person simulated mixtures (see below).

The study follows the policies of the Danish National Centre for Ethics and the Danish Research Ethics Committees (https://nationaltcenterforetik.dk/english (accessed on 15 August 2025)) and complies with the rules of the General Data Protection Regulation (Regulation (EU) 2016/679).

### 2.2. Construction of Simulated Mixtures

Simulated mixtures were generated from FASTQ files of 20 Danish individuals using the in-house Python script MixtureSimulator v1.0 (https://github.com/dk-cph-ri-rga/MixtureSimulator). The samples were chosen from two Danish cohorts, one sequenced with the Ion AmpliSeq™ MH-74 Plex Research Panel (hereafter: the MH-74 plex) [21] and the other with the ForenSeq™ DNA Signature Prep Kit (hereafter: the ForenSeq Kit) [22]. The 10 individuals per cohort presented little read count variance between each other and total read counts close to the cohort’s average (370,000 reads for the MH-74 plex and 220,000 reads for the ForenSeq Kit). MixtureSimulator v1.0, using the package BioPython v1.84 [26], produced FASTQ files of simulated mixtures with user-selected contributor proportions, number of individuals per mixture, and read count of the final FASTQ file. The script shuffled and randomly selected reads from FASTQ files of each single source sample, e.g., 333,000 reads from sample A and 37,000 reads from sample B, to simulate a 9:1 mixture with 370,000 reads.

A total of 180 two-person mixtures were simulated for each of the MPS panels, 45 per mixture ratio (19:1, 9:1, 3:1, and 1:1). Additionally, 168 three-person mixtures were created, 56 per mixture ratio (14:5:1, 14:3:3, and 9:9:2).

### 2.3. PCR Amplification and MPS Library Building of Real Mixtures

The master mix for the PCR amplification of the 72 mixtures (36 in duplicate) was prepared using 4 µL of 5X Ion AmpliSeq™ HiFi Mix (Thermo Fisher Scientific), 10 µL of Ion AmpliSeq™ Custom MH Panel (Thermo Fisher Scientific) containing the primers for the Ion AmpliSeq™ MH-74 Plex Research Panel, and 5 µL of nuclease-free water. An amount of 19 µL of the master mix was placed in a 96-well PCR plate, followed by 1 µL of each sample (DNA input 1 ng). All samples were typed in duplicate. A VeritiPro™ 96-Well Thermal Cycler (Thermo Fisher Scientific) was used to perform the PCR with the following conditions: 99 °C, 2 min; (99 °C, 15 s; 60 °C, 4 min) × 24 times; hold at 10 °C.

DNA libraries were prepared from amplicons using the Precision ID Library Kit (Thermo Fisher Scientific) and an Ion Express™ Barcode X Kit (Thermo Fisher Scientific). Libraries were purified using Agencourt AMPure XP Reagents (Agencourt, Beverly, MA, USA) and quantified with the Qubit 4.0 Fluorometer using the Qubit dsDNA HS Assay Kit (Thermo Fisher Scientific).

### 2.4. DNA Sequencing of Real Mixtures

The 72 mixtures were equally divided into two runs. The libraries were diluted with low TE Buffer to a final concentration of 100 pM and pooled in 1.5 µL Eppendorf tubes. Twenty-five microliters of each pool were loaded in the Ion Chef™ System (Thermo Fisher Scientific) according to the protocol. The Ion Chef ™ System carried out template preparation and loading of the Ion 530™ chips using the Ion S5™ Precision ID Chef Reagents (Thermo Fisher Scientific). Sequencing was performed on the HID Ion GeneStudio™ S5 System (Thermo Fisher Scientific) using the Ion S5™ Precision ID Sequencing Reagents (Thermo Fisher Scientific) and Ion 530™ Chip Kits (Thermo Fisher Scientific).

### 2.5. Data Analysis of Mixtures

**Figure 1:**
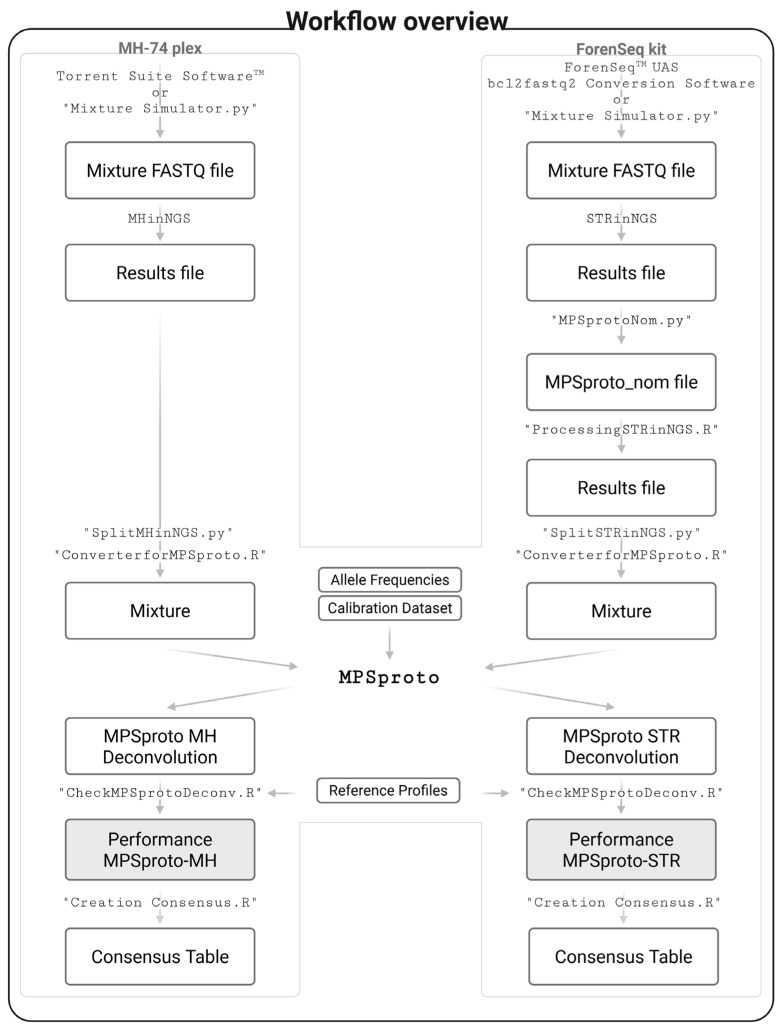
Flowchart of data analysis workflows for the different data types (MH mixtures on the left and STR mixtures on the right). In-house scripts are denoted with quotation marks, while software is referenced without. The probabilistic genotyping software employed for the mixture deconvolution is represented in bold. Created with Biorender.com.

The overall workflow for the data analysis is shown in Figure 1.

The two workflows shared the same probabilistic genotyping software for the deconvolution but were otherwise independent. The workflows were used for both simulated and real mixtures.

#### 2.5.1. MH Mixtures

The MH data analysis (Figure 1) involved (1) downloading of the run results from the Torrent Suite™ Software 5.10.1 as FASTQ files or use of the FASTQ files from MixtureSimulator v1.0, (2) analysis of the samples using the Python application MHinNGS v1.0 [23], and (3) deconvolution of the mixtures using MPSproto [27] (see below).

MHinNGS v1.0 [23] is a specifically designed tool for microhaplotype analysis in single-end sequencing data, and it requires a reference genome in FASTA format and a configuration file (Supplementary File S1). Analysis was performed using the most recent version of the MH-74 plex configuration file [28] with some additional modifications. These involved (1) the addition of 15 ‘ignored positions’ [23] in three loci (MH03KK-150, MH09KK-033rs144629057rs138516013, and MH09KK-153), and (2) manual adjustments to the allele names of MH09KK-152rs10746697, MH13KK-213rs2152726rs2152727, and MH22KK-061.

The output from MHinNGS was a unique result file, which was modified with the Python script “SplitMHinNGS” using the package pandas v2.2.0 [29]. This script separated the different samples into individual Excel files. Afterwards, each file was processed using the script “Converter for MPSproto”, which imported the file into R and modified it using the packages readxl v1.4.3 [30], dplyr v1.1.4 [31], stringr v1.5.1 [32], tidyr v1.3.0 [33], reshape2 v1.4.4 [34], and writexl v1.4.2 [35]. The output of the latter code was an Excel file containing the alleles in a format compatible with MPSproto.

#### 2.5.2. STR Mixtures

The STR data analysis (Figure 1) involved (1) downloading of BCL files from the ForenSeq™ Universal Analysis Software (UAS) (Verogen, San Diego, CA, USA) and converting them to FASTQ files using the bcl2fastq2 Conversion Software v2.20.0.422 (Illumina, San Diego, CA, USA), (2) generating simulated mixtures using MixtureSimulator v1.0 or directly using the FASTQ files of the real mixtures, (3) analysing the mixtures using the STR sequence analysis tool STRinNGS v2.1 [24], and (4) deconvolution of the mixtures using MPSproto [27] (see below).

STRinNGS v2.1 analyses single-end read sequencing files, using a reference genome in FASTA format and a configuration file with locus-specific information. A configuration file was developed and optimized for the ForenSeq™ DNA Signature Prep Kit (Supplementary File S2). The locus D22S1045 was not included in the configuration file because it was highly imbalanced [22]. Manual adjustments in the genotype calls were sometimes necessary for the loci D5S818, D10S1248, PentaD, and PentaE due to heterozygote imbalance. Following genotyping, the allele nomenclature was converted into a format compatible with MPSproto. The output file from STRinNGS was processed using a Python script named “MPSprotoNom”. This script utilized the packages pandas v2.2.0 and numpy v1.16.4 [36] to convert the allele names into the MPSproto bracketed format.

Finally, the output Excel file was processed by the Python script “SplitSTRinNGS”, which used the package pandas v2.2.0 to separate the different samples into individual Excel files. The files were then processed using the script “Converter for MPSproto”, which adjusted the mixtures into a format compatible with MPSproto.

#### 2.5.3. MPSproto Deconvolution

MPSproto v0.9.7 [27] was used to deconvolute both real and simulated mixtures. First, MPSproto required a calibration step performed with a dataset of single-source samples. Two different calibration datasets were prepared for each assay, one for the real mixtures and another for the simulated ones. Preliminary tests of MPSproto indicated better performance when the number of reads of the mixture resembled the reads in the profiles used for calibration. Thus, two datasets were prepared for MH deconvolution: one based on 50 Somali individuals [28] with read counts that were similar to the real mixtures and one based on 50 Danish individuals [21] with read counts that were similar to the simulated mixtures. The profiles were processed with the script “Creation Calibration Dataset for MPSproto” with the aid of the packages readxl v1.4.3, dplyr v1.1.4, tidyr v1.3.0, stringr v1.5.1, and writexl v1.4.2. The script (1) directly modified the output file from MHinNGS, (2) retained the alleles and read count data, and (3) distinguished between predicted alleles and noise at every locus. The script assigned a “dose” value to every allele: 0 to noise alleles, 1 to alleles of a heterozygous locus, and 2 to the allele of a homozygous locus. The dose was primarily used during MPSproto’s calibration.

Two other datasets of 50 Danish individuals [22] were prepared for the STRs. The profiles were converted with the bcl2fastq2 Conversion Software, analysed with STRinNGS, and processed with “MPSprotoNom”, “ProcessingSTRinNGS”, and “Creation calibration Dataset for MPSproto”.

All the calibration datasets were loaded in R and processed with MPSproto. The software iterated through these broad datasets and applied an analytical default threshold of 11 reads. MPSproto used the dose values in the calibration datasets to (1) evaluate the locus efficiency, (2) obtain per-locus information about stutters (for STRs) and noise reads, and (3) store this information in a definitive RDS file. One primary change to the original script [27] was made to the calibration step of the MH-74 plex, because these loci do not present stutter artefacts. A new function, “getNoiseData”, was created to evaluate noise reads and later store the calibrated model in an RDS file.

Second, MPSproto required allele frequencies of the corresponding MPS panels. The MH-74 plex allele frequencies were calculated based on 141 Danish individuals. The individuals included in the simulated and real mixtures were added to the 125 profiles used for the allele frequency calculations by Tomas et al. [21], to ensure that all of their alleles were observed in the population. The ForenSeq Kit allele frequencies were taken from the study of 363 Danish individuals [22]. As the profiles used to create the simulated mixtures were taken from that cohort, the alleles were already observed in the population. The allele nomenclature from Hussing et al. [22] was converted to a bracketed format compatible with MPSproto by the in-house script “NomenclatureConverter” and the packages readxl v1.4.3, dplyr v1.1.4, stringr v1.5.1, and tidyr v1.3.0.

Third, the number of contributors (NOC) in the mixture was set to two or three, and the two hypotheses H_p_ and H_d_ in the LR = P (E|H_p_)/P(E|H_d_) were specified. H_p_ and H_d_ were the same and defined as “The sample consists of DNA from two unknown individuals” (or three in three-person mixtures) when MPSproto deconvoluted the mixtures, and the results were matched against the known profiles. Every mixture deconvolution was assessed with the script “Check MPSproto deconvolution” using the packages readxl v1.4.3, tidyr v1.3.0, openxlsx v4.2.7.1 [37], dplyr v1.1.4, and tibble v3.2.1 [38]. This script evaluated the deconvolution by comparing the proposed major and minor contributor profiles generated by MPSproto with the known single-source profiles. The script “Check MPSproto deconvolution” assigned “Correct” to loci that were correctly deconvoluted and “Wrong” to loci with at least one wrongly predicted allele. Each prediction came with an assigned probability calculated by MPSproto. All proposed genotypes with a probability lower than the default value of 0.9 and genotypes with a proposed drop-out were assigned as “Undetermined”.

The two independent deconvolutions of the real MH and STR mixtures were merged into a final “Consensus deconvolution” with the aid of the script “Creation Consensus” using the packages readxl v1.4.3 and writexl v1.4.2. Only identical deconvolutions were accepted, while differently deconvoluted loci were classified as “Undetermined”.

The 360 simulated two-person mixtures were also deconvoluted 50 times each with another H_p_: “The sample consists of DNA from an unknown contributor and the suspect”, while H_d_: “The sample consists of DNA from two unknown individuals” remained the same. A total of 50 different single-source profiles were chosen as ‘suspect’, one of which was the true minor contributor to the mixture. Similarly, 50 deconvolutions for each of the 336 simulated three-person mixtures were carried out with the latter H_p_ and H_d_, adjusted for the correct number of contributors. Out of the 50 deconvolutions per mixture, the ‘suspect’ was only once a true contributor to the mixture. The true contributor was the minor contributor in 9:9:2 mixtures, the major in 14:3:3 mixtures, and the intermediate in 14:5:1 mixtures (the intermediate contributor is the contributor with the second most reads in a three-person mixture).

## 3. Results

### 3.1. MPSproto Analyses of Simulated Two-Person Mixtures

A total of 360 two-person mixtures were simulated from previously generated single-source profiles obtained with either the MH-74 plex [21] or the ForenSeq Kit [22]. The simulated mixtures had different proportions of the major and minor contributors, ranging from 1:1 mixtures up to 19:1 mixtures. The deconvolutions performed by MPSproto were compared to the known profiles, and the performance is summarised in Table 1 (MHs) and Table 2 (STRs). Overall, the deconvolution error rate was 0.4% (112/26,640) for the MH-74 plex and 2.3% (214/9360) for the ForenSeq Kit.

The deconvolutions of the major contributor profiles were almost 100% accurate in the most unbalanced mixtures (19:1 and 9:1), and MPSproto made no wrong genotype calls in either MH or STR simulated data when using the default probability threshold of 0.9. In the more evenly balanced mixtures (3:1), the number of correctly deconvoluted loci for the major contributors was lower, and seven and eight wrong genotypes were called in five different loci for MH and STR mixtures, respectively. These wrong calls were scrutinized and seemed to fall into three categories (examples in Supplementary Table S2): (1) A skewed heterozygote balance from the single-source sequencing data was transferred to the simulated mixture and affected the deconvolution of the mixed profile. This was evident in five of the eight 3:1 STR mixtures with wrong genotype calls. (2) Relative differences in locus read depths between the two single-source samples, combined with the random selection of reads from these samples, resulted in a mixture, where the defined ratio (e.g., 3:1) did not resemble the actual ratio of reads from the two contributors. There were two examples of that in the STR mixtures and three examples in the MH mixtures. (3) A combination of the two categories above.

**Figure 2:**
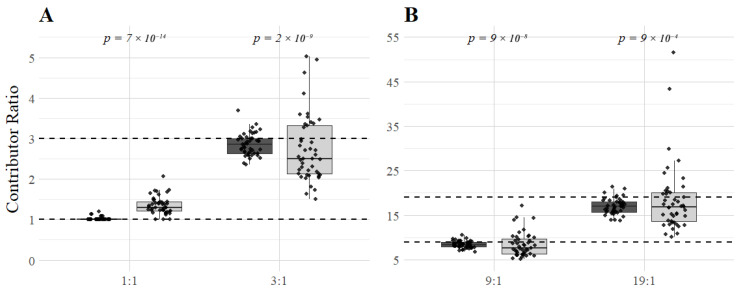
Boxplots representing the variance of the contributor proportions estimated by MPSproto in the 180 MH (dark grey) and 180 STR (light grey) simulated mixtures across all mixture ratios (1:1 and 3:1 in (**A**), 9:1 and 19:1 in (**B**)). The black dashed lines represent the expected ratios (1, 3, 9, and 19, for the 1:1, 3:1, 9:1, and 19:1 mixtures, respectively). Each black dot represents the contributor proportion of a mixture, calculated as the proportion of the major contributor divided by the minor contributor. For the balanced mixtures, the contributor proportion was calculated as follows: read counts of the individual with the highest read counts/read counts of the individual with the lowest read counts. The *p*-values from Wilcoxon rank sum tests are shown at the top of the boxplots, in italics.

In the 1:1 mixtures of MH profiles, only loci with one detected MH allele were successfully deconvoluted (12.3% of the loci, approximately nine loci per contributor), and there were no wrong genotype calls. In loci with more than one detected allele, the probability threshold of 0.9 was not reached, and the deconvolution was deemed uncertain, as would be expected for 1:1 mixtures. This was in stark contrast to the 1:1 mixtures of STR profiles, where 21.9% of the STR loci were deconvoluted, resulting in 188 incorrect genotype calls (8%) in 22 different loci (Table 2). Incorrect genotype calls were identified in 20 of the 45 1:1 mixtures, and all of them had estimated contributor proportions that deviated from 1:1. MPSproto estimated the contributor proportions of the mixtures (Figure 2) and used them for deconvolution of the profile. When it was reasonably accurate (1:1 up to 1.25:1) for the 1:1 mixtures of STR profiles, very few genotype calls had a probability higher than 0.9 and were deemed acceptable. However, when the contributor proportion was estimated at 1.3:1 and up to 2:1, the number of genotype calls was higher (up to 44 in one 1:1 mixture), and one-third of these genotype calls were incorrect (examples in Supplementary Table S3).

The estimates of the contributor proportions for the 1:1 mixtures of MHs (Figure 2) were highly accurate (39 of the 45 MH mixtures were estimated at 1:1), whereas the contributor proportions of the STR 1:1 mixtures were not (only 3 of the 45 STR mixtures were estimated at 1:1), and this seemed to explain the differences in deconvolution accuracy between MH and STR 1:1 mixtures (Table 1 and Table 2). Statistical significance of the variation in contributor proportion estimates was assessed with a Wilcoxon rank sum test, since the data deviated from a normal distribution (Shapiro–Wilk test *p*-values were lower than 0.05 for most mixture proportions). The obtained *p*-values (Figure 2) confirmed that MPSproto estimated the contributor proportions in MH mixtures more accurately than the contributor proportions in STR mixtures across all mixture ratios.

The deconvolutions of the minor contributors had overall lower success rates than the major contributors (Table 1 and Table 2). However, the percentage of correctly deconvoluted genotypes was higher for MHs than for STRs. In the deconvolutions of MH loci, 104 wrong genotypes were observed in the minor contributor of 19:1, 9:1, and 3:1 mixtures. Most of these (65%) were allele drop-outs and seemed to originate from relatively small differences in read depths between the alleles, e.g., 1476 ACTT and 1113 ACTC reads in the MH09KK-020 example in Supplementary Table S4. In this example, the two individuals were heterozygous for the same alleles, and the ACTC allele was not assigned to the minor contributor by MPSproto. If the balance between the alleles had been more even, the genotype probability for the minor contributor would most likely have been lower than 0.9, and the result would have been undetermined. A total of 35 drop-ins were observed in the MH mixtures. Most of these (89%) were noise assigned to the minor contributor (example in Supplementary Table S4) and would most likely be identified as such by an experienced analyst. In only four incidents of MH drop-ins, an allele from the major contributor, with relatively many reads, was assigned to the minor contributor with a high probability.

For STRs, the deconvoluted genotypes of the minor contributors in 19:1 mixtures were few but correct. In contrast to the 19:1 mixtures of MHs, there were no drop-ins or drop-outs in the minor contributor, which may be explained by low genotype probabilities due to the presence of stutters from the allele(s) of the major contributor. In the 9:1 and 3:1 mixtures of STRs, 18 wrong genotypes were observed in eight different loci. The reason for the wrong genotype calls seemed to be the same as mentioned above for the major contributors: either the heterozygote balance was unusual in the major or minor contributor, or the difference in relative locus read depths was large.

### 3.2. Log_10_LR of Minor Contributors in Two-Person Mixtures

**Figure 3:**
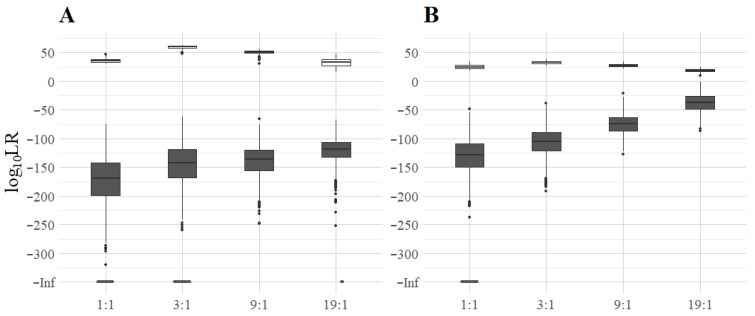
Log_10_LRs based on (**A**) the MH-74 plex and (**B**) the ForenSeq Kit. The white boxes represent the log_10_LR of the deconvolutions where the ‘suspect’ was in the mixture, whereas the grey ones represent deconvolutions where the ‘suspect’ was not in the mixture. The dark grey dots at the bottom (log_10_LR = −Inf) were obtained exclusively when the ‘suspect’ was not in the mixture (1344, 1067, and 2 times in the MH 1:1, 3:1, and 19:1 mixtures, respectively, and 116 times in the STR 1:1 mixtures).

A total of 18,000 deconvolutions were performed using the 360 simulated mixtures generated above, and LRs were calculated based on two hypotheses: H_p_, “The sample consists of DNA from an unknown contributor and the suspect” and H_d_, “The sample consists of DNA from two unknown individuals”. For each mixture of MHs or STRs, 50 different ‘suspects’ were tested, one of which was the minor contributor, whereas 49 of the ‘suspects’ did not contribute to the mixture (Figure 3).

In the 180 deconvolutions where the ‘suspect’ was indeed the true minor contributor to the mixture, the LRs ranged from 10^15^ to 10^63^ for the MH-74 plex and 10^9^ to 10^37^ for the ForenSeq Kit. The largest LRs were obtained in 3:1 mixtures and the lowest LRs in 19:1 mixtures with both assays. The overall higher log_10_LR values in the MH data could be explained by the higher number of loci present in the assay (74) compared to the ForenSeq Kit (26).

In the 17,640 deconvolutions where the ‘suspect’ was not a contributor to the mixture, the LRs ranged from 10^−62^ to 10^−320^ for the MH-74 plex and from 10^−2^ to 10^−238^ for the ForenSeq Kit. Furthermore, MPSproto returned LRs of −Inf (LR = 0) for 2413 deconvolutions with the MH-74 plex and 116 with the ForenSeq Kit (Figure 3). These extremely low values were obtained in 1:1 (1344 times), 3:1 (1067 times), and 19:1 (2 times) mixtures for the MH-74 plex, and only in 1:1 mixtures for the ForenSeq Kit. The mean log_10_LR values for non-contributors increased as the mixture became more imbalanced for both assays. This was most evident for the STR mixtures, where the mean LR was 10^−37^ in 19:1 mixtures and the LR > 10^−6^ for 22 of the 2205 deconvolutions.

### 3.3. MPSproto Analyses of Real Mixtures

In addition to the analyses of simulated mixtures, 72 real two-person mixtures (36 mixtures typed in duplicate) sequenced with the MH-74 plex and 18 real two-person mixtures (9 mixtures typed in duplicate) sequenced with the ForenSeq Kit [7] were analysed with the workflow in Figure 1 to demonstrate that real mixtures could also be deconvoluted.

Estimates of the contributor proportions calculated by MPSproto (Supplementary Table S5) were fairly accurate except for a few of the mixtures, where the estimated ratio varied by a factor of three compared to the actual ratio. The deconvolutions performed by MPSproto were compared to the known profiles, and the performance is summarised in Table 3 (MHs) and Table 4 (STRs).

Overall, the number of correctly deconvoluted genotypes was lower for the real mixtures (Table 3 and Table 4) than for the simulated ones (Table 1 and Table 2). This was especially evident for the minor contributors’ genotypes. However, the overall number of wrongly deconvoluted genotypes was also lower (0.05% for the MH-74 plex and 0.2% for the ForenSeq Kit).

In the major contributors, there were only two errors. One in a 3:1 mixture typed with the MH-74 plex, where the read depth was low overall, and one in a 6:1 mixture typed with the ForenSeq Kit that led to drop-in alleles in both the minor and major contributor (Supplementary Table S6). In the latter example, a stutter from the major contributor was not recognized and, instead, assigned to the minor contributor. However, it was not immediately clear why MPSproto would make this mistake and return a genotype probability for the major contributor of one, when the heterozygote balance was almost 10:1. Five wrong genotype calls in the minor contributors were observed in mixtures typed with the MH-74 plex. These errors were all drop-ins of noise assigned as one of the minor contributor alleles. The contributor proportions of all the 1:1 mixtures were accurately estimated (Supplementary Table S5), and only loci with one detected allele were deconvoluted for both MHs and STRs. Thus, the many wrong genotype calls observed for the simulated 1:1 mixtures of STRs were not observed in the two real 1:1 mixtures.

None of the wrong genotype calls were reproduced in the duplicate typing of the real mixtures. Thus, the consensus profiles from the two deconvolutions were 100% accurate. Supplementary Table S7 shows the number of deconvoluted loci and the Random Match Probability (RMP) of the major and minor contributor consensus profiles. The MH profiles of the minor contributors consisted of 3–27 genotypes, and the RMP ranged from 9.4 × 10^−1^ to 2.2 × 10^−19^, whereas the STR profiles of the minor contributors had only one deconvoluted locus (TPOX) and an RMP of 3.5 × 10^−1^. The major contributors of the MH mixtures had RMPs ranging from 2 × 10^−2^ in more balanced mixtures to 3.7 × 10^−70^ in the most unbalanced ones, whereas the major contributors of the STR mixtures had RMPs ranging from 3.5 × 10^−1^ to 3 × 10^−38^. The values were higher than those in the minor contributors due to the higher number of deconvoluted loci (up to 74 for MHs and 26 for STRs).

### 3.4. MPSproto Analyses of Simulated Three-Person Mixtures

A total of 336 three-person mixtures with three different contributor proportions were simulated as described for the two-person mixtures and deconvoluted with MPSproto. One set of mixtures (9:9:2 mixtures) consisted of two major contributors with equal contributions and one minor contributor. Another set of mixtures (14:3:3 mixtures) consisted of one major contributor and two minor contributors with equal contribution, whereas the third set (14:5:1 mixtures) consisted of one major contributor, one intermediate contributor, and one minor contributor. The deconvolutions performed by MPSproto were compared to the known profiles, and the performance is summarised in Table 5 (MHs) and Table 6 (STRs).

The percentages of correctly deconvoluted genotypes were higher for MHs than for STRs in all mixture proportions, and the overall deconvolution error rate was lower for MHs (0.6% (142/24,864)) compared to STRs (2.3% (197/8736)).

In the 9:9:2 mixtures, the minor contributor was genotyped correctly in an average of 16 MH loci and 5 STR loci, whereas the deconvolutions of the two major contributors were much less successful. A total of 56 wrong genotype calls were observed in the deconvoluted MH profiles from the major contributors. In 42 out of 56 9:9:2 MH mixtures, the major contributor proportions were correctly estimated as equal (Supplementary Figure S1). Only loci with one major allele were correctly deconvoluted, and the major allele was assigned to both major contributors. However, when the major contributors were estimated as unequal (from 1.1:1 to 1.2:1), loci with two or more alleles with high read counts were deconvoluted and, in some cases, falsely assigned to the two major contributors (example in Supplementary Table S8). For the STRs, the number of wrong genotype calls in the two major contributors (a total of 136) was higher than the number of correct ones (example in Supplementary Table S8). The wrong genotype calls were observed in 22 9:9:2 STR mixtures, and in all of these, the contributor proportion of the two major contributors was not accurately predicted as equal (ranged from 1.25:1 to 1.6:1) (Supplementary Figure S1). If the two major contributors were estimated to contribute almost equally (from 1:1 to 1.25:1), there were no wrong genotype calls in the two major contributors, which was very similar to the observations in the 1:1 two-person mixtures of STR profiles.

In the 14:3:3 mixtures, the deconvoluted profile of the major contributor was almost complete, and the numbers resembled the results from the major contributors of 3:1 two-person mixtures (Table 1 and Table 2). In contrast, very few loci were deconvoluted for the two minor contributors of the 14:3:3 mixtures, which was expected since they contributed equally to the mixture. No wrong MH genotype calls were detected in the minor contributors, and only nine wrong genotype calls were observed in the STRs (example in Supplementary Table S9). Accordingly, the contributor proportions of the two minor contributors (Supplementary Figure S1) were highly accurate for MH mixtures and reasonably accurate (from 1:1 to 1.25:1) for most STR mixtures.

In the 14:5:1 mixtures, many correct genotype calls were made for the major and intermediate contributors in both assays. However, the number of wrong genotype calls in the intermediate contributors was higher, especially for the STR mixtures. Overall, the deconvolutions of major and intermediate contributors in 14:5:1 mixtures resembled the results from the major and minor contributors of 3:1 two-person mixtures (Table 1 and Table 2). For the minor contributors to the 14:5:1 mixtures, the average number of correctly deconvoluted loci was 9.5 and 0.6 with the MH-74 plex and the ForenSeq Kit, respectively. This was a little lower than observed for the minor contributors of 19:1 two-person mixtures.

### 3.5. Log_10_LR of Minor Contributors in Three-Person Mixtures

**Figure 4:**
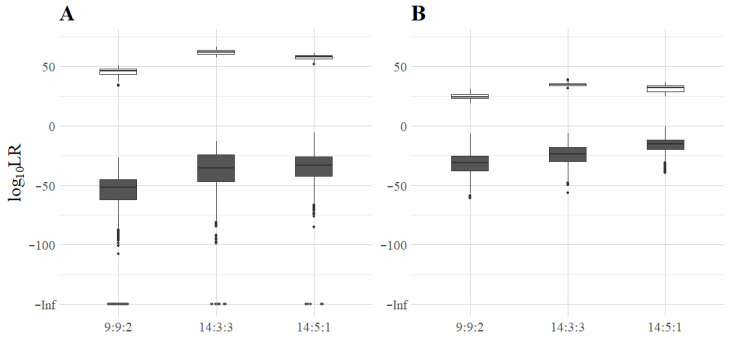
Log_10_LRs based on (**A**) the MH-74 plex and (**B**) the ForenSeq Kit. The white boxes represent the log_10_LRs of the deconvolutions where the ‘suspect’ was part of the mixture (the minor contributor of 9:9:2 mixtures, the major contributor of 14:3:3 mixtures, and the intermediate contributor of 14:5:1 mixtures), whereas the grey ones represent deconvolutions where the ‘suspect’ was not in the mixture. The dark grey dots at the bottom of the graphs (log_10_LR = −Inf) were obtained exclusively when the ‘suspect’ was not in the mixture (190, 8, and 6 times in the 9:9:2, 14:3:3, and 14:5:1 MH mixtures, respectively).

A total of 16,800 deconvolutions were performed using the 336 simulated three-person mixtures, and LRs were calculated based on two hypotheses: H_p_, “The sample consists of DNA from two unknown contributors and the suspect” and H_d_, “The sample consists of DNA from three unknown individuals”. For each mixture of MHs or STRs, 50 different ‘suspects’ were used, one of which was a true contributor, whereas 49 of the ‘suspects’ did not contribute to the mixture. In the 9:9:2 mixtures, the true contributor was always the minor contributor; in the 14:3:3 mixtures, only the major contributor was used as ‘the suspect’, while in the 14:5:1 mixtures, the true contributor was the intermediate contributor. In the 336 deconvolutions, where the ‘suspect’ was indeed a true contributor to the mixture, the MH-74 plex obtained LRs between 10^34^ and 10^66^, while the ForenSeq Kit LRs ranged from 10^19^ to 10^39^ (Figure 4).

The highest LRs were obtained for the major contributor of the 14:3:3 mixtures and the lowest for the minor contributor of the 9:9:2 mixtures with both assays. In the 16,464 deconvolutions, where the ‘suspect’ was not in the mixture, the LRs were < 10^−6^ for 99.5% (16,374/16,464) of the simulated mixtures. However, in 89 of the 14:5:1 STR profiles and one of the 14:5:1 MH profiles, the LR was > 10^−6^. For six non-contributors to 14:5:1 STR profiles, the LR was even higher than 10^−3^.

## 4. Discussion

In this study, a uniform workflow for mixture deconvolution of sequenced MHs and STRs was developed using a combination of in-house scripts and the probabilistic genotyping software MPSproto [25]. A total of 360 two-person and 336 three-person mixtures, created with the Python script MixtureSimulator v1.0 from previously generated single-source sequencing data [21,22], and 90 real two-person mixtures [7,21] were analysed with the workflow. The deconvolution ability was evaluated by (1) counting the number of correctly and wrongly deconvoluted loci in the profiles, (2) analysing the accuracy of the estimated contributor proportions, and (3) calculating LRs for true contributors and non-contributors to the mixtures.

Overall, the deconvoluted MH profiles were more complete than the deconvoluted STR profiles, especially for the minor contributors to the mixtures, and the error rate of the deconvoluted genotypes was 4–5 times lower for MHs than for STRs. Furthermore, the estimated contributor proportions were more accurate for MHs than for STRs. These results clearly show that MHs are preferred over STRs for mixture deconvolution.

The wrong genotype calls in the deconvoluted profiles were studied at length and could be categorised into groups. In the major contributors, the wrong genotype calls were mainly caused by skewed heterozygote balances or differences in locus read depths between the single-source samples that were used to generate the simulated mixtures. The former reason was mostly observed in STRs, which correlates with previous analyses of heterozygote balances in sequencing data [7,9,39], and may be related to the fact that long STR alleles usually have fewer reads than short STR alleles and that stutters may contribute to the allele imbalance. We speculate that the latter reason may be an unforeseen consequence of the mixture simulation. In a real mixture, it is unlikely that DNA from one person is preferentially amplified over DNA from another person in the same reaction, unless the DNA from one of the people is damaged. However, in the simulated mixtures, the amplicons/sequencing reads are generated in independent reactions, and locus balance variations may be more likely. We speculate that the wrong genotype calls caused by the differences in locus read depths between the single-source samples are mostly artificial and would not appear in a real mixture. Unfortunately, the results from the real mixtures analysed in this work could not be used to confirm this assumption, because there were few real mixtures and almost no wrong genotype calls in the deconvoluted profiles (only seven). The wrong genotype calls in the minor contributors were mainly caused by relatively small variations in read depth between alleles that led to the drop-out of an allele, or drop-in of noise, or, rarely, an allele from the major contributor. These results resemble those of previous studies of mixtures [8,9,10,13,16,17].

The LRs calculated on the simulated mixtures with MPSproto correctly identified all true contributors (LR > 10^15^ for MHs and LR > 10^9^ for STRs) and excluded the non-contributors (LR < 10^−6^ for MHs and LR < 0.2 for STRs). In the ENFSI Guideline for Evaluative Reporting in Forensic Science [40], LRs < 10^−5^ and LRs > 10^5^ provide very strong support for correctly excluding and including contributors to the mixture, respectively. The ForenSeq Kit did not provide very strong support for excluding the non-contributors in several 19:1 and 14:5:1 mixtures, as opposed to the MH-74 plex. However, it must be considered that the LR differences between the kits may be partly explained by the number of loci in each assay (74 MH and 26 STR markers).

A stark difference in performance between MHs and STRs was found in mixtures with equal contributors (1:1, 9:9:2, and 14:3:3). The profiles of the equal contributors can only be correctly deconvoluted if they are homozygous for the same allele. If the two equal contributors have a total of two, three, or four distinct alleles, it is not possible to deconvolute the mixture, and the probabilistic genotyping software should return a low probability for the deconvoluted profiles. This also happened for MH and STR mixtures, where the contributor proportions were correctly estimated. However, when the contributor ratio was estimated at 1.25:1 or higher, the probability for the deconvoluted genotype was frequently higher than the default threshold set for trustworthy deconvolution (*p* > 0.9). This happened frequently for STR mixtures, and approximately one-third of these genotypes were incorrect. For MH mixtures, the estimation of contributor proportions was more accurate, and only a few wrong genotype calls were observed in these mixtures. We speculate that the presence of stutters and different-sized STR alleles, leading to heterozygote imbalances, had a negative impact on the estimation of contributor proportions. Reads from a stutter with the same sequence as an allele increase the heterozygote imbalance and may resemble the data present in a mixture with other contributor proportions. In the 14:3:3 mixtures of STRs, where the equal contributors were both minor contributors, the contributor proportions were more accurate (Supplementary Figure S1), and almost no wrong genotype calls were made in the minor contributors. This may be explained by the fact that most of the stutters originating from the alleles of the two minor contributors to these mixtures were removed by the noise filter of the analysis software (STRinNGS) and thus were not imported into MPSproto.

Mixture interpretation is an essential part of forensic genetic casework, and the results presented here show that future (sequencing) assays developed for this purpose should include loci with low levels of noise and low variation in heterozygote and locus balances. MPSproto assigned some noise to minor contributor profiles, and it may be possible to optimize the probabilistic genotyping software with the purpose of improving its performance during the mixture deconvolution process. Different noise thresholds of MPSproto or de-noising software following the mixture genotyping could be tested to identify and remove noisy reads. Alternatively, the sequencing analysis software could be designed to identify and remove these reads. In this work, we used MHinNGS [23] and STRinNGS [24] to analyse the sequencing data, and these software packages have functions that are used to eliminate or ignore noise. This is highly useful for analyses of single-source samples but comes with the risk of eliminating relevant information or biological variation. Stutters cannot be eliminated as easily, and the read depth imbalances caused by STR length differences will also remain as a problem that needs to be solved by the probabilistic genotyping software. Future assays for mixture interpretation would benefit from the introduction of MHs to the core set of forensic genetic loci, and the MH working group under the International Society of Forensic Genetics is currently working towards this end (Podini et al., publication in preparation). MHs have proved to be highly useful for relationship testing [11,13,20,21,28,41,42], and the MH-74 plex was recently implemented as a supplementary panel for more complicated kinship cases in our laboratory [21,28]. In this work, we demonstrate that MHs are superior to STRs for mixture deconvolution and thus also have a place in crime casework.

## Figures and Tables

**Table 1:** MPSproto deconvolution success rates (in percent of the number of genotypes) for the MH-74 plex mixtures.

Mixtures ^1^	Major Contributor			Minor Contributor		
	Correct	Wrong	Undetermined ^2^	Correct	Wrong	Undetermined ^2^
19:1	99.9%	0%	0.1%	22.3%	1.8%	75.9%
9:1	99.8%	0%	0.2%	36.1%	0.6%	63.3%
3:1	93%	0.2%	6.8%	75.6%	0.7%	23.7%
1:1	12.3%	0%	87.7%	12.3%	0%	87.7%

^1^ Every row is the average of 45 different mixtures sharing the same contributor proportions. ^2^ The deconvolution was evaluated as uncertain (*p* < 0.9) by MPSproto.

**Table 2:** MPSproto deconvolution success rates (in percent of the number of genotypes) for the ForenSeq Kit mixtures.

Mixtures ^1^	Major Contributor			Minor Contributor		
	Correct	Wrong	Undetermined ^2^	Correct	Wrong	Undetermined ^2^
19:1	100%	0%	0%	7.3%	0%	92.7%
9:1	99.7%	0%	0.3%	30.2%	0.4%	69.4%
3:1	82%	0.7%	17.3%	58.9%	1.1%	40%
1:1	14%	8%	78%	13.9%	8%	78.1%

^1^ Every row is the average of 45 different mixtures sharing the same contributor proportions. ^2^ The deconvolution was evaluated as uncertain (*p* < 0.9) by MPSproto.

**Table 3:** MPSproto deconvolution (in percent of the number of genotypes) of the real MH-74 plex two-person mixtures.

Mixtures ^1^	Major Contributor			Minor Contributor		
	Correct	Wrong	Undetermined ^2^	Correct	Wrong	Undetermined ^2^
29:1	99.5%	0%	0.5%	5.9%	0%	94.1%
9:1	88.7%	0%	11.3%	20.1%	0.2%	79.7%
3:1	78.5%	0.2%	21.3%	35.8%	0.2%	64%
2:1	24.6%	0%	75.4%	20.3%	0%	79.7%
1:1	19.2%	0%	80.8%	19.2%	0%	80.8%
1:2	23.3%	0%	76.7%	21.4%	0%	78.6%
1:3	60.8%	0%	39.2%	34.1%	0.2%	65.7%
1:9	99%	0%	1%	23.2%	0.3%	76.5%
1:29	99.3%	0%	0.7%	6.4%	0%	93.6%

^1^ Every row is the result of the average of 8 mixtures with the same contributor proportion typed with the MH-74 plex. ^2^ The deconvolution was evaluated as uncertain (*p* < 0.9) by MPSproto.

**Table 4:** MPSproto deconvolution (in percent of the number of genotypes) of the real ForenSeq Kit two-person mixtures.

Mixtures ^1^	Major Contributor			Minor Contributor		
	Correct	Wrong	Undetermined ^2^	Correct	Wrong	Undetermined ^2^
25:1	96.2%	0%	3.9%	0%	0%	100%
12:1	100%	0%	0%	1.9%	0%	98.1%
6:1	86.6%	1.9%	11.6%	5.8%	1.9%	92.3%
3:1	53.9%	0%	46.1%	11.5%	0%	88.5%
1:1	3.8%	0%	96.2%	3.8%	0%	96.2%
1:3	9.6%	0%	90.4%	5.8%	0%	94.2%
1:6	92.3%	0%	7.7%	3.8%	0%	96.2%
1:12	96.1%	0%	3.9%	0%	0%	100%
1:25	100%	0%	0%	0%	0%	100%

^1^ Every row is the result of the average of 2 mixtures with the same contributor proportion typed with the ForenSeq Kit. ^2^ The deconvolution was evaluated as uncertain (*p* < 0.9) by MPSproto.

**Table 5:** Deconvolution performance of the MH-74 plex three-person mixtures (in percent of the number of genotypes).

Mixtures ^1^	Contributor 1			Contributor 2			Contributor 3		
	Correct	Wrong	Undetermined ^2^	Correct	Wrong	Undetermined ^2^	Correct	Wrong	Undetermined ^2^
9:9:2	12.7%	0.8%	86.5%	12.7%	0.5%	86.8%	22.2%	0.3%	77.5%
14:3:3	94.9%	0.4%	4.7%	9.7%	0%	90.3%	9.7%	0%	90.3%
14:5:1	88.6%	0.4%	11%	71.6%	0.6%	27.8%	12.9%	0.3%	86.8%

^1^ Every row is the result of the average of 56 different mixtures with the same contributor proportion typed with the MH-74 plex. ^2^ The deconvolution was evaluated as uncertain (*p* < 0.9) by MPSproto.

**Table 6:** Deconvolution performance of the ForenSeq Kit three-person mixtures (in percent of the number of genotypes).

Mixtures ^1^	Contributor 1			Contributor 2			Contributor 3		
	Correct	Wrong	Undetermined ^2^	Correct	Wrong	Undetermined ^2^	Correct	Wrong	Undetermined ^2^
9:9:2	1.86%	5.2%	92.9%	1.8%	4.1%	94.1%	17.7%	0.6%	81.7%
14:3:3	85%	0.6%	14.4%	0.3%	0.3%	99.4%	0.4%	0.3%	99.3%
14:5:1	77.2%	0.7%	22.1%	48.2%	1.6%	50.2%	2.5%	0.1%	97.4%

^1^ Every row is the result of the average of 56 different mixtures with the same contributor proportion typed with the ForenSeq Kit. ^2^ The deconvolution was evaluated as uncertain (*p* < 0.9) by MPSproto.

## Data Availability

The original contributions presented in this study are included in the article/Supplementary Materials. Further inquiries can be directed to the corresponding author.

## Supplementary Materials

The following supporting information can be downloaded at: https://www.mdpi.com/article/10.3390/genes16091105/s1, Figure S1: Variance of contributor ratios calculated by MPSproto in three-person mixtures; Table S1: Samples and volumes used to create the real MH-74 plex two-person mixtures; Table S2: Two examples of wrong deconvolutions of 3:1 mixtures; Table S3: Three examples of wrong deconvolutions of 1:1 mixtures; Table S4: Three examples of wrong deconvolutions of 19:1 mixtures; Table S5: Contributor ratios of the real two-person mixtures calculated by MPSproto; Table S6: Examples of wrong deconvolutions in D2S441 that occurred in both duplicates of a real 6:1 ForenSeq mixture; Table S7: Random Match Probability of the major and minor contributor consensus profiles from the real mixtures; Table S8: Two examples of wrong deconvolutions of 9:9:2 mixtures; Table S9: One example of a wrong deconvolution of 14:3:3 mixtures. Supplementary File S1: Configuration file for the MH74 plex; Supplementary File S2: Configuration file for the ForenSeq kit.
