# Supplementary material for: Mixture Deconvolution with Massively Parallel Sequencing Data: Microhaplotypes Versus Short Tandem Repeats
Source: Genes (Basel). 2025 Sep 18;16(9):1105. doi: 10.3390/genes16091105 (PMC12470170; doi:10.3390/genes16091105)
Supplement: Supplementary file 1 [file genes-16-01105-s001.zip › Supplementary Figure.pdf]

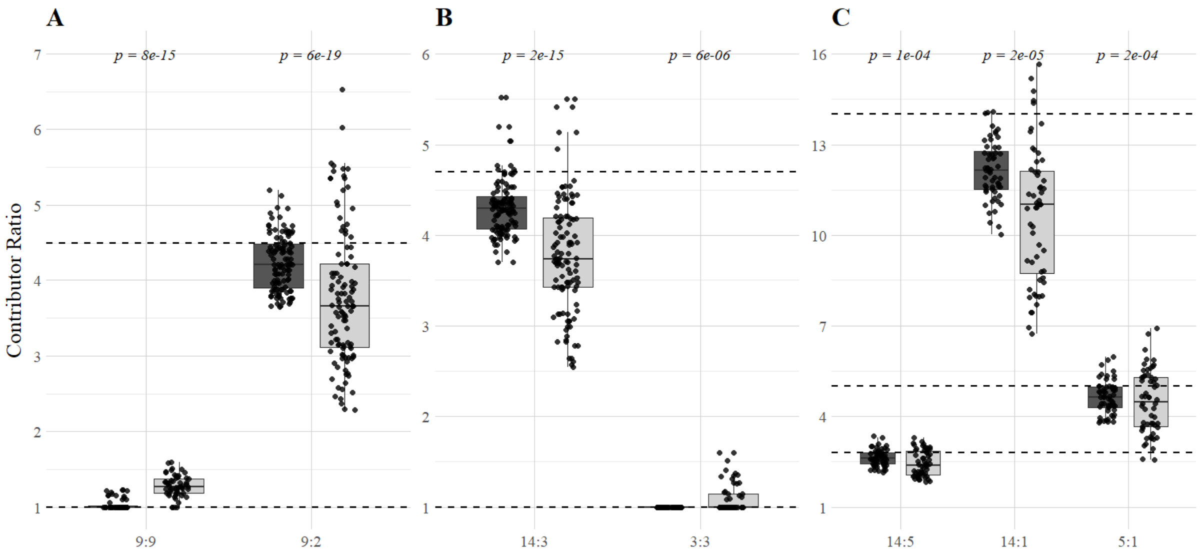

Supplementary Figure S1. Boxplot representing the variance of the contributor ratios calculated by MPSproto in the 168 MH (dark grey) and 168 STR (light grey) three-person mixtures (9:9:2 in A, 14:3:3 in B, and 14:5:1 in C). For the 9:9:2 mixtures, the mixture ratio 9:2 includes the ratios observed for both major contributors with the minor. Similarly, the mixture ratio 14:3 includes the observed ratio of the major contributor with both minor contributors. The black dashed lines represent the expected ratios. For the balanced mixtures, the contributor proportion was calculated as: read counts of the individual with the highest read counts/read counts of the individual with the lowest read counts. The p-values from Wilcoxon Rank Sum Tests are shown at the top of the boxplots, in italics.
